# Complete Genome Sequence of *Dyella thiooxydans* ATSB10, a Thiosulfate-Oxidizing Bacterium Isolated from Sunflower Fields in South Korea

**DOI:** 10.1128/genomeA.00573-16

**Published:** 2016-06-23

**Authors:** Kyeong Hwangbo, Yurry Um, Hee Chung, Jemin Yoo, Ki Yoon Kim, Munusamy Madhaiyan, Tong Min Sa, Yi Lee

**Affiliations:** a Department of Industrial Plant Science & Technology, Chungbuk National University, Cheongju, South Korea; b Department of Herbal Crop Research, National Institute of Horticultural and Herbal Science, Rural Development Administration, Eumseong, South Korea; c Department of Environmental and Biological Chemistry, Chungbuk National University, Cheongju, South Korea; d Biomaterials and Biocatalysts Group, National University of Singapore, Singapore

## Abstract

*Dyella thiooxydans* ATSB10 (KACC 12756^T^ = LMG 24673^T^) is a thiosulfate-oxidizing bacterium isolated from rhizosphere soils of sunflower plants. In this study, we completely sequenced the genome of *D. thiooxydans* ATSB10 and identified the genes involved in thiosulfate oxidation and the metabolism of aromatic intermediates.

## GENOME ANNOUNCEMENT

*Dyella thiooxydans* is a novel species that was discovered from rhizosphere soil of sunflower (*Helianthus annuus* L.) cultivated in the Republic of Korea (1). This type strain, *D. thiooxydans* ATSB10 (KACC 12756^T^ = LMG 24673^T^), is a thiosulfate-oxidizing bacterium. Also, this strain is a Gram-negative, aerobic, motile, rod-shaped, and facultative chemolithoautotroph. This species was described as a new species in the genus *Dyella* by Anandham et al. (1) and stimulated plant growth and root elongation of canola by rock phosphate solubilization. This study will provide an understanding of the specific genes related to plant growth of thiosulfate-oxidizing bacteria.

Genomic DNA of *D. thiooxydans* ATSB10 was extracted using the PowerSoil DNA isolation kit (Mo Bio, CA). By using a combination of PacBio and Illumina MiSeq sequencing, we generated a total of 81,855 and 13,081,160 reads, respectively, with 1,070× coverage of the genome. The reads were *de novo* assembled by PacBio SMRT Analysis Suite version 2.3 (PacBio) and CLC Genomics Workbench 7.5, which resulted in a single contig. This generated contig was merged using the CodonCode aligner (CodonCode Corp.). Error correction and quality check for the resulting contig were performed by manual curation. Gene prediction in the assembled genome was performed using the Integrated Microbial Genomes-Expert Review (IMG-ER) platform (2, 3). Prediction of open reading frames (ORFs) was conducted and compared using SEED (4), Clusters of Orthologous Groups (COG) (5), EzTaxon-e database bacterial rRNA profiles (6), and Pfam (the Protein Families database) (7, 8) for gene annotation. To improve genome annotation quality and rRNA and tRNA gene prediction, gene function analysis was performed with the Rapid Annotations using Subsystems Technology (RAST) server databases (9). The gene caller Glimmer 3.02, RNAmmer 1.2 (10), and tRNAscan-SE 1.23 (11) were also used to identify rRNA genes and tRNA genes, respectively. The genome of *D. thiooxydans* consists of a 4,227,172-bp circular chromosome with a G+C content of 70.0%. A total of 3,862 protein-coding genes of average length 976 bp were predicted, along with 6 rRNA and 49 tRNA genes.

The analysis of the *D. thiooxydans* ATSB10 genome revealed the presence of two sulfite oxidase genes (ATSB10_00650 and ATSB10_11060) involved in the tetrathionate-intermediate (S4-I) pathway for thiosulfate oxidation, and two rhodanese genes (*sseA*, ATSB10_12220; and *pspE*, ATSB10_33570). In addition, three glucose dehydrogenase genes (*gcd*; ATSB10_10580, ATSB10_32210, and ATSB10_32660) involved in rock phosphate solubilization were detected (12). *D. thiooxydans* has a total of 74 genes involved in the aromatic pathway, including 21 genes involved in the metabolism of central aromatic intermediates and 15 genes of a peripheral pathway for the catabolism of aromatic compounds. Moreover, we identified the genes involved in sulfur compound biosynthesis, such as *S*-adenosyl-l-methionine (a key intermediate in methionine metabolism), coenzyme A, lipoic acid, and biotin. This study is the first fully sequenced and annotated genome of the species *D. thiooxydans* ATSB10 and will provide valuable information about microbial taxonomy, systematics, and biotechnological applications.

### Nucleotide sequence accession number.

The complete chromosome sequence has been deposited at DDBJ/EMBL/GenBank under accession no. CP014841. The version described in this paper is the first version.
